# Genetic deletion or TWEAK blocking antibody administration reduce atherosclerosis and enhance plaque stability in mice

**DOI:** 10.1111/jcmm.12221

**Published:** 2014-01-30

**Authors:** Cristina Sastre, Valvanera Fernández-Laso, Julio Madrigal-Matute, Begoña Muñoz-García, Juan A Moreno, Carlos Pastor-Vargas, Patricia Llamas-Granda, Linda C Burkly, Jesús Egido, Jose L Martín-Ventura, Luis M Blanco-Colio

**Affiliations:** a Lab. Patología Vascular, IIS-Fundación Jiménez DíazMadrid, Spain; b Biogen IdecCambridge, MA, USA

**Keywords:** TWEAK, atherosclerosis, inflammation, stability

## Abstract

Clinical complications associated with atherosclerotic plaques arise from luminal obstruction due to plaque growth or destabilization leading to rupture. Tumour necrosis factor ligand superfamily member 12 (TNFSF12) also known as TNF-related weak inducer of apoptosis (TWEAK) is a proinflammatory cytokine that participates in atherosclerotic plaque development, but its role in plaque stability remains unclear. Using two different approaches, genetic deletion of TNFSF12 and treatment with a TWEAK blocking mAb in atherosclerosis-prone mice, we have analysed the effect of TWEAK inhibition on atherosclerotic plaques progression and stability. Mice lacking both TNFSF12 and Apolipoprotein E (TNFSF12^−/−^ApoE^−/−^) exhibited a diminished atherosclerotic burden and lesion size in their aorta. Advanced atherosclerotic plaques of TNFSF12^−/−^ApoE^−/−^ or anti-TWEAK treated mice exhibited an increase collagen/lipid and vascular smooth muscle cell/macrophage ratios compared with TNFSF12^+/+^ApoE^−/−^ control mice, reflecting a more stable plaque phenotype. These changes are related with two different mechanisms, reduction of the inflammatory response (chemokines expression and secretion and nuclear factor kappa B activation) and decrease of metalloproteinase activity in atherosclerotic plaques of TNFSF12^−/−^ApoE^−/−^. A similar phenotype was observed with anti-TWEAK mAb treatment in TNFSF12^+/+^ApoE^−/−^ mice. Brachiocephalic arteries were also examined since they exhibit additional features akin to human atherosclerotic plaques associated with instability and rupture. Features of greater plaque stability including augmented collagen/lipid ratio, reduced macrophage content, and less presence of lateral xanthomas, buried caps, medial erosion, intraplaque haemorrhage and calcium content were present in TNFSF12^−/−^ApoE^−/−^ or anti-TWEAK treatment in TNFSF12^+/+^ApoE^−/−^ mice. Overall, our data indicate that anti-TWEAK treatment has the capacity to diminish proinflamatory response associated with atherosclerotic plaque progression and to alter plaque morphology towards a stable phenotype.

## Introduction

Atherosclerosis is a chronic disease affecting large arteries that involves the formation of plaques containing vascular and inflammatory cells, lipids and extracellular matrix 1. Its complication arises through obstruction of the arterial lumen, leading to an ischaemic event. Two different mechanisms have been proposed for this process: plaque growth leading to vessel stenosis, and/or formation of unstable plaques that acutely rupture, leading to an occlusive thrombus formation 2. Despite great advances in the treatment of atherosclerosis, there is still a high mortality rate in these patients.

Tumour necrosis factor ligand superfamily member 12 (TNFSF12) gene encodes a protein known tumour necrosis factor-like weak inducer of apoptosis (TWEAK) that is a proinflammatory cytokine belonging to TNF superfamily. TWEAK induces, through its receptor fibroblast growth factor-inducible 14 (Fn14), a high number of physiological and pathological processes depending on cell type and environment 3. Within the vasculature, TWEAK is expressed in non-atherosclerotic arteries 4,5, while Fn14 is expressed at low levels or is absent in healthy tissues 4. Interestingly, both TWEAK and Fn14 are expressed in human atherosclerotic plaques colocalizing with vascular smooth muscle cells (VSMC) and macrophages 4. TWEAK is implicated in different processes associated with atherogenesis such as inflammation 6, proliferation and migration of VSMC 7, thrombosis 8 and angiogenesis 7,8. We demonstrated that systemic administration of TWEAK aggravates atherosclerotic plaque development in the aortic root of apolipoprotein E (ApoE) knockout mice 9. Furthermore, inhibition of Fn14 by an Fn14-Fc decoy protein reduces lesion size in atherosclerotic mice 10.

Certain characteristics of human atherosclerotic plaques, such as a greater lesion size, decreased VSMC content, increased macrophage number, and evidence of healed silent plaque rupture, are associated with instability and rupture 11 and similar phenotypic features are observed in unstable brachiocephalic atherosclerotic lesions of the ApoE^−/−^ mice 12, suggesting that mouse lesions mimic at least some of the processes that culminate in plaque rupture in humans 13. Brachiocephalic arteries from mice present features of plaque instability such as lateral xanthomas, buried caps, medial erosion, large necrotic core and calcification that are more evident than in the aortic root.

In the present article, we aimed to fully assess the role of TWEAK in the formation of an unstable plaque phenotype. To do so we used ApoE^−/−^ mice with genetic deficiency of TNFSF12 and analysed early and advanced atherosclerotic lesions present in their aortic root. Moreover, we explored whether anti-TWEAK mAb administration could protect mice from vascular inflammation and progression to an unstable plaque phenotype. In addition, we have tested the effect of TNFSF12 deletion or anti-TWEAK treatment on features related to plaque instability in brachiocephalic arteries of ApoE^−/−^ mice.

## Materials and methods

### Animals

Female ApoE^−/−^ mice (#002052; Jackson Laboratory, Bar Harbor, ME, USA ) were crossed with male TNFSF12^−/−^ mice (generously provided by Biogen Idec. Cambridge, MA, USA; both on the C57BL/6 background), and the progeny bred back to ApoE^−/−^ mice for seven generations followed by intercrossing to obtain the double knockout (TNFSF12^−/−^ApoE^−/−^) and their littermate control (TNFSF12^+/+^ApoE^−/−^) and TWEAK-heterozygous (TNFSF12^+/−^ApoE^-/^) mice. Male mice were fed on a standard chow diet during the experiment. To study the effect of TNFSF12 deletion on early atherosclerotic lesions, TNFSF12^+/+^ApoE^−/−^ (*N* = 9), TNFSF12^+/−^ ApoE^−/−^ (*N* = 7) and TNFSF12^−/−^ApoE^−/−^ (*N* = 9) were killed at 24 weeks of age.

To study the effect of anti-TWEAK treatment on established atherosclerotic lesions, 24 weeks-old TNFSF12^+/+^ApoE^−/−^ mice (*N* = 27) were randomized in three groups; mice injected i.p. with saline (*N* = 9), anti-TWEAK mAb (10 mg/kg twice a week; *N* = 10) or an irrelevant isotype-matched control IgG specific for Hen egg lysozyme (10 mg/kg twice a week; *N* = 8) during 16 weeks. In addition, TNFSF12^+/−^ ApoE^−/−^ (*N* = 8) and TNFSF12^−/−^ApoE^−/−^ (*N* = 9) mice were included to analyse the effect of TNFSF12 deletion on advanced atherosclerotic plaques. The blocking anti-mouse TWEAK mAb (clone P2D10) was generated in TNFSF12^−/−^ mice 14. All mice were killed at 40 weeks of age (Fig. S1).

All mice were maintained under barrier conditions. Water and normal laboratory diet was available *ad libitum*. Mice were anesthetized by intraperitoneal injection of ketamine (100 mg/kg) and xylazine (15 mg/kg). Blood was obtained through retro-orbital puncture. Hearts were perfused with sterile saline *via* the left ventricle at physiological pressure and aortas were dissected.

Cholesterol was tested in serum samples Amplex Red Cholesterol assay kit (Invitrogen, Carlsbad, CA, USA). HDL-c, LDL-c/VLDL-c and triglyceride concentrations were measured in serum with HDL and LDL/VLDL cholesterol assay kit and triglyceride quantification kit, respectively (Abcam, Cambridge, England).

The housing and care of animals and all the procedures carried out in this study were strictly in accordance with the Directive 2010/63/EU of the European Parliament and were approved by the Institutional Animal Care and Use Committee of IIS-Fundación Jimenez Diaz.

### En face of aorta

Atherosclerotic lesions were quantified by en face analysis of the whole aorta and by cross-sectional analysis of the aortic root and the innominate artery. For en face preparations, the aorta was opened longitudinally, from the heart to the iliac arteries, while still attached to the heart and major branching arteries in the body. The aorta (from the heart to the iliac bifurcation) was then removed and was ‘pinned out’ on a white wax surface in a dissecting pan using stainless steel pins 0.2 mm in diameter. After overnight fixation with 4% paraformaldehyde and a rinse in PBS, the aortas were immersed for 6 min. in a filtered solution containing 0.5% Sudan IV, 35% ethanol and 50% acetone; and destained in 80% ethanol. The Sudan IV–stained aortas were photographed and were used for quantification of atherosclerotic lesions.

### Aortic root and brachiocephalic artery morphometric analysis

Brachiocephalic arteries and hearts containing aortic roots were carefully dissected and frozen in OCT (Sakura, AJ Alphen aan den Rijn, the Netherlands). Aortic roots were sectioned at 5 μm thickness beginning proximally at the first evidence of the aortic valves at their attachment site of aorta. Sections were stained with Oil red O/haematoxylin and haematoxilin at 100 μm intervals from 0 to 1000 μm distal to the site. Maximal lesion area was calculated for each mouse by averaging the values for three sections. The individual maximal lesion areas were further averaged to determine the maximal lesion area for each group.

Brachiochephalic arteries were serially sectioned in 5 μm thickness from the aortic root to the right subclavian artery. For morphometric analysis, sections of each brachiocephalic artery were stained with modified Russell-Movat pentachrome (Movat) at 90 μm intervals from 0 to 450 μm distal to the aortic root. The frequency of plaque instability features in each Movat-stained section was evaluated (five slides per animal, 40–50 slides per group), including the following: thin fibrous cap (defined as <3 cell layers), large necrotic core (defined as occupying >50% of the volume of the plaque), intraplaque haemorrhage (defined as the presence of red blood cells within the plaque and confirmed by TER-119 immunostaining), medial enlargement/erosion (defined as the replacement of the normal media by plaque components), lateral xanthomas (defined as the presence of aggregates of macrophage-derived foam cells situated on the lateral margins of the plaques) and the presence of buried caps (signature of silent plaque rupture). Calcification was analysed on the basis of positive staining with the von Kossa stain. These parameters were recorded as binary outcome, and the frequency per lesion for each animal was determined with a maximum possible of 100%.

### Immunohistochemical analysis

Immunohistochemical analysis was done in all animals included in each group. Picrosirius red staining was performed for analysis of collagen content by measuring birefringence to plane-polarized light. Immunohistochemistry was carried out as previously described 9. Primary antibodies were the macrophage marker Mac-3 (M3/84; BD Pharmingen, San Jose, CA, USA), the foam cell marker adipophilin (GP41; Progen, Heidelberg, Germany), the smooth muscle cells marker smooth muscle actin (Clone 1A4; Sigma-Aldrich, Buchs SG, Switzerland), the T cell marker CD3 (IR503/IS503; DAKO, Glostrup, Denmark), the chemokines MCP-1 (sc-1785; Santa Cruz Biotechnology, Dallas, TX, USA) and RANTES (AB2109P; Chemicon-Merck Millipore, Billerica, MA, USA), tissue factor (TF; 4515; American Diagnostica Inc., Lexington, MA, USA), the red blood cell marker TER-119 (sc-19592; Santa Cruz Biotechnology) and the proliferation marker PCNA (sc-7907; Santa Cruz Biotechnology). Apoptosis in the aortic valve region was determined using the TUNEL method as recommended by the manufacturer (ApopTag Peroxidase In Situ Apoptosis Detection Kit, S7100; Millipore Ibérica, Madrid, Spain).

Donkey anti-goat biotin, donkey anti-rabbit biotin, goat anti-rat biotin (Amersham-GE Healthcare, Little Chalfont, UK) and goat anti-guinea pig biotin (Abcam) were used as secondary antibodies. ABComplex/HRP was then added and sections were stained with 3,3-diaminobenzidine (DAKO), counterstained with haematoxylin and mounted in Pertex (Kaiser's glycerol gelatine, Merck Millipore, Billerica, MA, USA). Sections were counterstained with Carazzi haematoxylin. Incubation without primary antibodies and/or irrelevant species- and isotype-matched immunoglobulins was used as a negative control for all immunostaining. Computer-assisted morphometric analysis was performed with the Image-Pro Plus software (version 1.0 for Windows). The threshold setting for area measurement was equal for all images. Samples from each animal were examined in a blinded manner. Results were expressed as% positive area *versus* total area of lipid (Oil red/O), Collagen, Mac-3, adipophilin, alpha-smooth muscle actin, MCP-1, RANTES and TF and as positive cells per square millimeters of lesion of CD3, PCNA and TUNEL.

### Southwestern histochemistry

This technique was developed to detect *in situ* the distribution and DNA-binding activity of transcriptional factors 15. Nuclear factor kappa B (NF-κB) consensus oligonucleotide from the promoter of RANTES was digoxigenin labelled with a 3′-terminal transferase (Roche, Basel, Switzerland). OCT-embedded tissue sections were fixed in 0.5% paraformaldehyde and incubated with 0.1 mg/ml DNase I. The DNA binding reaction was performed by incubation with 50 pmol of the labelled DNA probe in buffer containing 0.25% BSA and 1 μg/ml poly (dI-dC). The sections were then incubated with alkaline phosphatase-conjugated antidigoxigenin Ab, and colorimetric detection was performed as described 15. Preparations without probe were used as negative controls, and an excess of unlabelled probe was used to test the specificity of the technique. Results are expressed as% positive area *versus* total area.

### *In situ* zymography

To estimate matrix metalloproteinases (MMP) gelatinase activity *in situ*, we incubated 5-μm sections using gelatin conjugated with quenched fluorescein (DQ-gelatin; Invitrogen, Carlsbad, CA, USA) as a substrate, which requires cleavage by gelatinolytic enzymes to become fluorescent. DQ-gelatin is a substrate only recognized by two gelatinases, MMP-2 and MMP-9. In brief, DQ-gelatin (1 mg/ml in H_2_O) was mixed 1:10 with 1% low-melting agarose (BioWithaker, Basel, Switzerland). This mixture (30 μl) was added on top of each section, coverslipped and gelled at 4°C. After 24 hrs incubation at 37°C, samples were examined under confocal fluorescence microscopy (Leica, Heerbrugg, Switzerland). Zymographic images were acquired using identical shutter conditions. The mean of fluorescence intensity of each cross-section, excluding the media area because of medial elastin filament autofluorescence, was measured. The specificity of gelatinase activity was determined by incubation of DQ-Gelatin in the presence of the broad-spectrum MMP-inhibitors ilomastat (10 μmol/l) and phenanthroline (0.5 mmol/l), for 24 hrs.

### Protein array

Soluble serum concentrations of 20 cytokines (IL-1α, IL-1β, IL-2, IL-3, IL-4, IL-5, IL-6, IL-9, IL-10, IL-12, IL-13, IL-17, M-CSF, TNF-α, KC, MCP-1, RANTES, VEGF and GM-CSF) were measured using a protein array following the manufacturer's instructions (Quantibody Mouse Cytokine Array 1; RayBiotech Inc., Norcross, GA, USA). Detection of signals was performed through use of a scanner equipped with Cy3 excitation/emission wavelength (555/565 nm; GenePix Professional 4200A, Genepix-Molecular Devices, Sunnyvale, CA, USA). Microarray analysis software was used to data extraction, as well as for quantitative data analysis. TNF-α, IL-1α, IL-3, IL-4, IL-12 and M-CSF concentrations were undetectable in serum samples from mice included in our animal model.

### RNA extraction and real-time PCR

RNA from total aorta of mice was obtained by TRIzol method (Life Technologies, Carlsbad, CA, USA) and quantified by absorbance at 260 nm in duplicate. Two microgram of total RNA was reverse transcribed at 37°C for 50 min. in a 20-μl reaction mix containing 200 U Moloney murine leukaemia virus RT (Invitrogen), 100 ng of random primers and 40 U RNase Inhibitor (Invitrogen). Reactions were terminated by heating to 95°C for 5 min., and samples were stored at −70°C. Real-time PCR was performed on a TaqMan ABI 7700 Sequence Detection System using heat-activated TaqDNA polymerase (Amplitaq Gold; Life Technologies). The following PCR primers and TaqMan probes were purchased from Life technologies and optimized according to the manufacturer's protocol: 18S, TWEAK (Mm00839900_m1), MCP-1 (Mm00441242_m1) and RANTES (Mm01302428_m1). After an initial hold of 2 min. at 50°C and 10 min. at 95°C, the samples were cycled 40 times at 60°C for 15 sec. and 60°C for 60 sec. 18S rRNA served as housekeeping gene and was amplified in parallel with the genes of interest. The expression of target genes was normalized to housekeeping transcripts. The amount of target mRNA in samples was estimated by the 2CT relative quantification method.

### Statistics

Statistical analysis was performed using SPSS 11.0 statistical software. Data are expressed as mean ± SD and due to abnormal data distribution, we selected the nonparametric Mann–Whitney *U*-test for non-paired data sets. Two-way anova test followed by Bonferroni posthoc correction was used for multiple group comparisons. A probability value of 0.05 was considered to be statistically significant.

## Results

### TNFSF12 deficiency reduces atherosclerotic plaque size and increases the plaque collagen/lipid ratio

To determine the role of TWEAK in atherosclerotic lesions, mice lacking both TNFSF12 and ApoE (TNFSF12^−/−^ApoE^−/−^), TNFSF12 heterozygous (TNFSF12^+/−^ApoE^−/−^) and single knockout controls (TNFSF12^+/+^ApoE^−/−^) were fed with a chow diet and tissue was harvested at 24 and 40 weeks, for study of early and advanced lesions, respectively (Fig. S1). TNFSF12^+/−^ApoE^−/−^ mice expressed around 40–50% less aortic TWEAK mRNA than TNFSF12^+/+^ ApoE^−/−^ mice (Fig. S2D). No significant differences were observed in metabolic measures such as bodyweight or lipid levels between the different groups (Fig. S2) or in aortic Fn14 mRNA expression (Fig. S2D).

**Figure 1:**
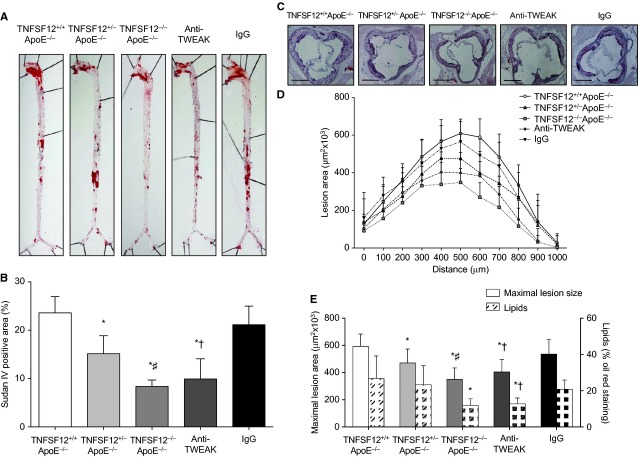
Tumour necrosis factor ligand superfamily member 12 (TNFSF12) deletion or anti-TNF-related weak inducer of apoptosis mAb administration reduced vascular damage in 40 weeks-old ApoE KO mice. (A) Representative pinned-out en face aorta preparations and (B) quantification of atherosclerosis from 40 weeks-old mice stained with Sudan-IV. Values shown are mean ± SD (*N* = 4–5 per group). *P < 0.001 *versus* TNFSF12^+/+^ApoE^−/−^; #*P* < 0.05 *versus* TNFSF12^+/−^ApoE^−/−^; †*P* < 0.01 *versus* control IgG. (C) Representative Oil-Red-O/Haematoxylin staining and (D) quantification of lesion area along the aortic root in 40 weeks-old mice. Values shown are mean ± SD of all animals included in each group. Scale bar, 200 μm. (E) Average of maximal lesions per group and quantification of Oil-Red-O staining in the aortic root of mice. Values shown are mean ± SD of all animals included in each group. *P < 0.001 *versus* TNFSF12^+/+^ApoE^−/−^; #*P* < 0.05 *versus* TNFSF12^+/−^ApoE^−/−^; †*P* < 0.05 *versus* control IgG.

In early lesions, we found a 44% reduction of the en face aorta lesion area in TNFSF12^−/−^ApoE^−/−^ mice compared with TNFSF12^+/+^ApoE^−/−^ mice (*P* < 0.05) while a 20% reduction was observed in the TNFSF12 heterozygous mice (Fig. S3A and B). TNFSF12^−/−^ApoE^−/−^ mice exhibited significantly reduced plaque size at the aortic root compared with control TNFSF12^+/+^ApoE^−/−^ mice (45% reduction; mean maximal lesion area; *P* < 0.01), while TNFSF12^+/−^ApoE^−/−^ mice showed a no significant 37% reduction (Fig. S3C–E). In the advanced lesions, both TNFSF12^−/−^ApoE^−/−^ and TNFSF12^+/−^ApoE^−/−^ mice showed significantly reduced en face aorta lesion area (64% and 36% reduction, *P* < 0.001; *P* < 0.05; respectively) and plaque size at the aortic root (41% and 21% reduction, *P* < 0.001; *P* < 0.05; respectively; Fig. 1A–E) as compared with TNFSF12^+/+^ApoE^−/−^ control mice.

**Figure 2:**
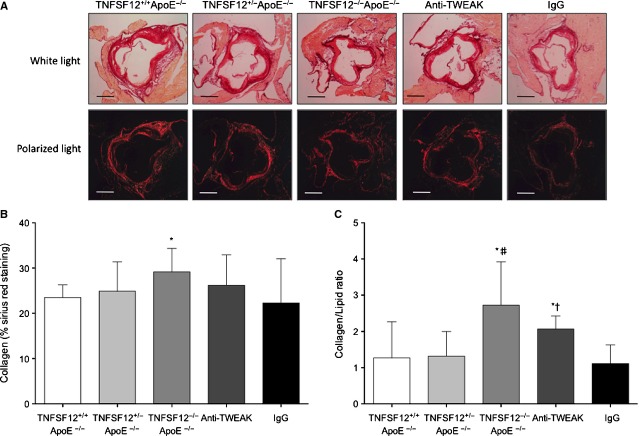
Tumour necrosis factor ligand superfamily member 12 (TNFSF12) deletion increased collagen content in the aortic root of 40 weeks-old ApoE KO mice. (A) Representative staining and (B) quantification of Sirius red in the aortic root of 40 weeks-old mice. Values shown are mean ± SD of all animals included in each group. *P < 0.05 *versus* TNFSF12^+/+^ApoE^−/−^. Scale bar, 200 μm. (C) Plaque stability was assessed by the collagen to lipid ratio. *P < 0.01 *versus* TNFSF12^+/+^ApoE^−/−^; #*P* < 0.01 *versus* TNFSF12^+/−^ApoE^−/−^; †*P* < 0.001 *versus* control IgG.

Whereas lipid deposits make plaques more prone to rupture, collagen fibres stabilize atherosclerotic plaques 16. Lipid content was defined as the percentage of Oil red/O staining area and plaque collagen content was defined as the percentage of Sirius Red area to total plaque area. No significant changes in lipid or collagen contents were observed in early lesions (data not shown). However, in advanced lesions, TNFSF12^−/−^ApoE^−/−^ exhibited reduced lipid content compared with TNFSF12^+/+^ApoE^−/−^ (*P* < 0.05; Fig. 1E). Moreover, collagen content was increased in TNFSF12^−/−^ApoE^−/−^ compared with TNFSF12^+/+^ApoE^−/−^ mice (*P* < 0.05; Fig. 2A and B). As a consequence, collagen/lipid ratio was increased in TNFSF12^−/−^ApoE^−/−^ mice compared with control mice (Fig. 2C).

### TNFSF12 deficiency reduces macrophages and foam cells and increases VSMC content at the aortic root

**Figure 3:**
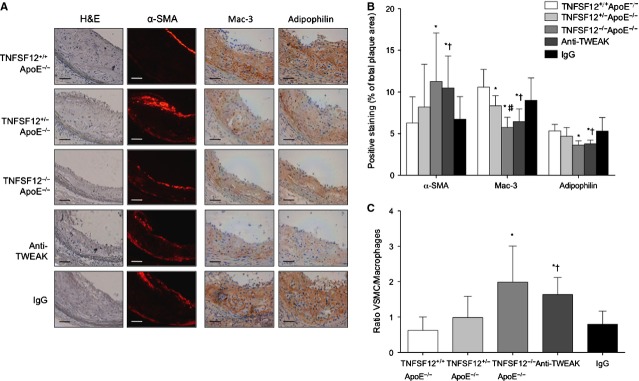
Tumour necrosis factor ligand superfamily member 12 (TNFSF12) deletion or anti-TNF-related weak inducer of apoptosis administration diminished macrophage content in advanced atherosclerotic plaques of ApoE KO mice. (A) Representative immunostaining and (B) quantification of α-SMA, Mac-3 and Adipophilin in aortic lesions from 40 week-old ApoE KO mice. Values shown are mean ± SD of all animals included in each group. *P < 0.05 *versus* TNFSF12^+/+^ApoE^−/−^; #*P* < 0.05 *versus* TNFSF12^+/−^ApoE^−/−^; †*P* < 0.05 *versus* control IgG. Scale bar, 50 μm. (C) Plaque stability was assessed by forming α-SMA to Mac-3 ratio. Values shown are mean ± SD. *P < 0.005 *versus* TNFSF12^+/+^ApoE^−/−^; †*P* < 0.001 *versus* control IgG.

As inflammation has a great impact on the vulnerability of plaques 17, we assessed the degree of inflammation in the aortic root of atherosclerotic mice. In early lesions, TNFSF12^−/−^ApoE^−/−^ showed a 53% decrease in the content of macrophages (Mac-3) and 34% in foam cells (Adipophilin) compared with control mice (*P* < 0.01 and *P* < 0.001; respectively; Fig. S4). Both parameters were also found decreased in heterozygous mice compared with controls, but only foam cells change was statistically significant (*P* = 0.005). In addition, TNFSF12^−/−^ApoE^−/−^ showed a 82% increment in VSMC content compared with TNFSF12^+/+^ApoE^−/−^ mice (*P* < 0.01; Fig. S4). In advanced atherosclerotic lesions TNFSF12^−/−^ApoE^−/−^ mice showed a 35% reduction in the content of T lymphocytes, 46% in macrophages and 32% in foam cells compared with controls (*P* < 0.001 for all types of cells; Fig. S5 and Fig. 3A). T cells and macrophage content were also diminished in heterozygous mice (*P* < 0.05). TNFSF12^−/−^ApoE^−/−^ mice exhibit a 79% increment in VSMC content compared with control mice (*P* < 0.01; Fig. 3). As a consequence, the VSMC/macrophage ratio was increased in the lesions of TNFSF12^−/−^ApoE^−/−^ mice, thus indicating a change to a more stable phenotype in advanced atherosclerotic lesions.

Changes in cellular composition observed in double knockout mice were not related with changes in plaque cellularity. The total number of cells was not changed in the different groups included in our study (Fig. S6). In addition, no changes in the amount of PCNA or TUNEL positive cells were observed between the different groups analysed (Fig. S6).

### TNFSF12 deficiency reduces proinflammatory response at the aortic root

**Figure 4:**
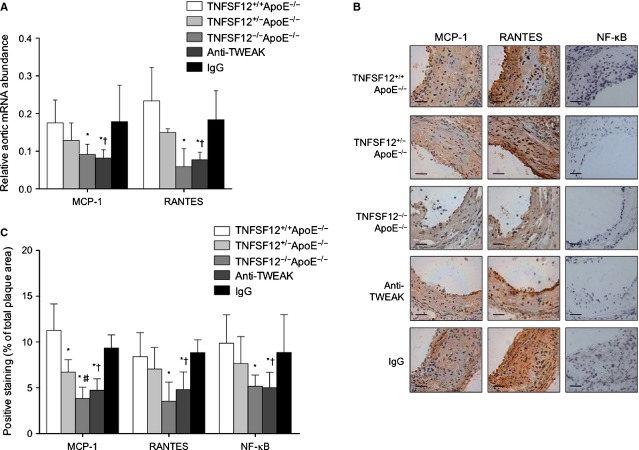
Tumour necrosis factor ligand superfamily member 12 (TNFSF12) deletion or anti-TNF-related weak inducer of apoptosis administration reduced pro-inflammatory chemokine expression and NF-κB activation in advanced atherosclerotic plaques of ApoE KO mice. (A) Quantitative real-time polymerase chain reaction analysis on MCP-1 and RANTES mRNA expression in total aorta of 40 weeks-old mice. Values shown are mean ± SD. *N* = 3–4 animals per group *P < 0.05 *versus* TNFSF12^+/+^ApoE^−/−^; #*P* < 0.05 *versus* TNFSF12^+/−^ApoE^−/−^; †*P* < 0.005 *versus* control IgG. (B) Representative immunostaining and (C) quantification of RANTES, MCP-1 and NF-κB in aortic lesions from 40 weeks-old ApoE KO mice. Values shown are mean ± SD of all animals included in each group. *P < 0.05 *versus* TNFSF12^+/+^ApoE^−/−^; #*P* < 0.005 *versus* TNFSF12^+/−^ApoE^−/−^; †*P* < 0.005 *versus* control IgG; scale bar, 50 μm.

To determine the mechanisms by which TNFSF12 deletion results in reduced macrophage content, we evaluated the expression of inflammatory chemokines that have a prominent role in the proinflammatory response associated with atherosclerotic plaque progression 18,19. MCP-1 and RANTES participate in the recruitment of monocytes/macrophages in atherosclerotic plaques 19 and their expression is controlled by the transcription factor NF-κB 20. Early lesions from TNFSF12^−/−^ApoE^−/−^ mice exhibited a diminished mRNA and protein expression of both chemokines and attenuated NF-κB activity when compared with their littermate controls (Fig. S7). The anti-inflammatory effect of TWEAK deficiency was more evident in advanced lesions with MCP-1 and RANTES mRNA and protein expression and NF-κB activation strongly diminished in TNFSF12^−/−^ApoE^−/−^ mice relative to controls (Fig. 4). In addition, TF participates in thrombus formation and its expression is also regulated by NF-κB. We have observed that TF expression was also decreased in both TNFSF12^−/−^ApoE^−/−^ and TNFSF12^+/−^ApoE^−/−^ treated mice compared with control animals (Fig. S8).

**Figure 5:**
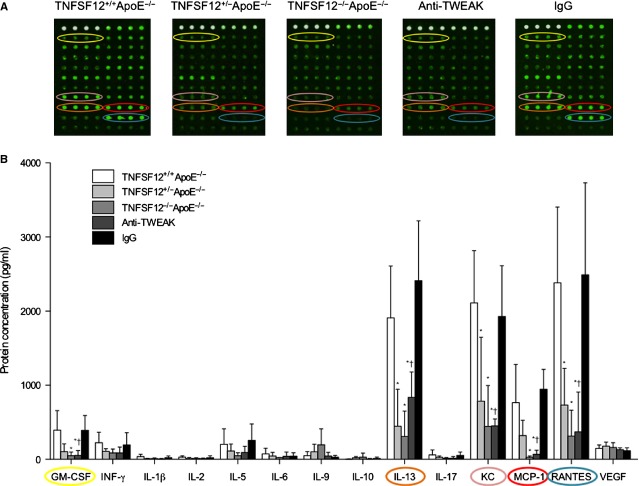
Tumour necrosis factor ligand superfamily member 12 (TNFSF12) deletion or anti-TNF-related weak inducer of apoptosis administration reduced cytokine serum concentrations in ApoE KO mice. (A) Representative cytokine array of serum from 40 weeks-old mice. Slides were scanned using a GenePix 4000A microarray scanner and image analysis was performed with GenePix Pro software (version 6.0). (B) Quantification of cytokines in serum from 40 weeks-old mice. Values shown are mean ± SD (*N* = 8 per group). *P < 0.05 *versus* TNFSF12^+/+^ApoE^−/−^; †*P* < 0.05 *versus* control IgG. GM-CSF: Granulocyte macrophage colony-stimulation factor; INF-γ: Interferon gamma; IL: Interleukins; KC: keratinocyte-derived chemokine; MCP-1: Monocyte chemoattractant protein 1; RANTES: regulated and normal T cell expressed and secreted.

To confirm changes in the systemic proinflammatory profile, protein array of 20 cytokines from serum of the different animal groups were done. Both MCP-1 and RANTES along with KC, GM-CSF and IL-13 concentrations were diminished in TNFSF12^−/−^ApoE^−/−^ compared with TNFSF12^+/+^ApoE^−/−^ mice (Fig. 5).

### Anti-TWEAK mAb treatment reduced lesion size and increased plaque stability at the aortic root

We further studied whether long anti-TWEAK treatment would protect against plaque progression from the early to the advanced stage. Twenty-four week-old TNFSF12^+/+^ApoE^−/−^ mice were treated with either anti-TWEAK mAb or control IgG (10 mg/kg/twice a week for both) during 16 weeks (Fig. S1). No significant differences were observed in metabolic measures such as bodyweight or lipid levels between the different groups (Fig. S2). As expected, no differences were observed between TNFSF12^+/+^ApoE^−/−^ and IgG-treated mice in any parameter analysed. Anti-TWEAK treated mice exhibited diminished en face aorta lesion area and plaque size compared with control mice (*P* < 0.05 for both; Fig. 1). Anti-TWEAK treated mice showed a reduction in lipid content at the aortic root compared with control mice (*P* < 0.05; Fig. 1E). No changes in collagen content were observed after anti-TWEAK treatment (Fig. 2). Collagen/lipid ratio was clearly increased in anti-TWEAK treated TNFSF12^+/+^ApoE^−/−^ compared with control mice (Fig. 2; *P* < 0.05), indicating that anti-TWEAK treatment increased plaque stability.

Anti-TWEAK treatment increased VSMC content (*P* < 0.001) and reduced T cells, macrophages and foam cells (*P* < 0.005 for CD3; *P* < 0.001 for Mac-3; *P* < 0.001 for Adipophilin) in aortic root lesions (Fig. 3 and Fig. S5). Vascular smooth muscle cell/Macrophage ratio was also increased in anti-TWEAK treated mice (Fig. 3C). Again, changes in cellular composition observed in anti-TWEAK treated mice were not related with changes in plaque cellularity. The total number of cells was not changed in anti-TWEAK compared with control mice (Fig. S6). In addition, no changes in the amount of PCNA or TUNEL positive cells were observed between the different groups analysed (Fig. S6).

MCP-1 and RANTES mRNA, protein expression and serum concentrations (*P* < 0.001 for all) and NF-κB activation (*P* < 0.01) were also diminished in anti-TWEAK treated mice relative to control mice (Fig. 5). In addition, TF expression was also reduced in anti-TWEAK treated mice compared with control mice (Fig. S8). All together, these data indicate that anti-TWEAK treatment diminished the inflammatory response and increased features of plaque stability in TNFSF12^+/+^ApoE^−/−^ mice.

### TNFSF12 deficiency or anti-TWEAK treatment improve features of brachiocephalic artery atherosclerotic plaque instability

The potential stabilizing effect of either TNFSF12 deletion or anti-TWEAK treatment was tested in brachiocephalic artery, in which advanced plaques present similar phenotypic features observed in unstable human lesions 13,21, and some of these features are more evident than in the lesion of aortic root. As atherosclerotic lesions of brachiocephalic arteries from 24 weeks-old mice represent an early step in atherosclerotic plaque development (mainly fatty streaks) 22, we therefore studied 40 week-old mice.

**Figure 6:**
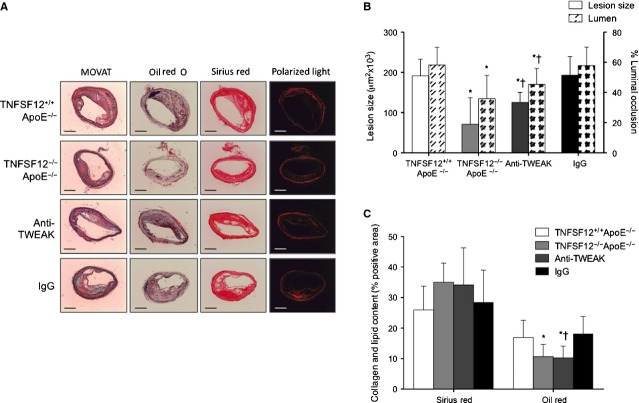
Tumour necrosis factor ligand superfamily member 12 (TNFSF12) deletion or anti-TNF-related weak inducer of apoptosis administration diminished atherosclerotic lesion size and% luminal occlusion in the brachiocephalic artery of ApoE KO mice. (A) Representative photographs of Movat's pentachrome sections, Sirius red–stained (collagen) and oil red O–stained (lipids) sections of the brachiocephalic artery from different groups; scale bar, 200 μm. (B) Average of maximal lesion area per group and percentage of luminal occlusion in the brachiocephalic artery. Values shown are mean ± SD of all animals included in each group. *P < 0.05 *versus* TNFSF12^+/+^ApoE^−/−^; †*P* < 0.01 *versus* control IgG. (C) Quantification of Sirius red and Oil red/O staining. Mean areas of lipid deposition and collagen content of all mice of one group are shown with SD. *P < 0.05 *versus* TNFSF12^+/+^ApoE^−/−^; †*P* < 0.05 *versus* control IgG.

Atherosclerotic lesion size and luminal occlusion were reduced in brachiocephalic artery of TNFSF12^−/−^ApoE^−/−^ (63% and 39%; respectively) compared with TNFSF12^+/+^ApoE^−/−^ controls (Fig. 6A and B). Mice treated with anti-TWEAK also showed a diminished plaque size and luminal occlusion (35% and 23%; respectively) at 40 weeks of age as compared with control mice (Fig. 6A and B).

**Figure 7:**
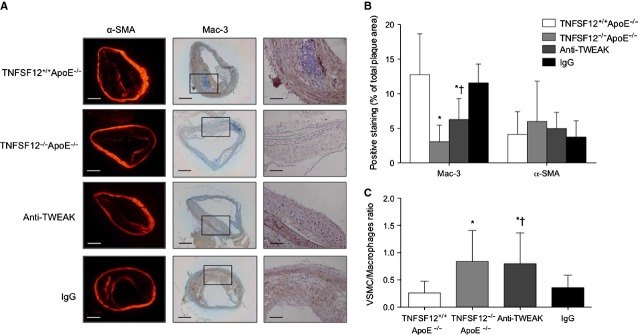
Tumour necrosis factor ligand superfamily member 12 (TNFSF12) deletion or anti-TNF-related weak inducer of apoptosis administration diminished macrophage content in brachiocephalic artery of ApoE KO mice. (A) Representative immunostaining and (B) quantification of α-SMA and Mac-3 in brachiocephalic lesions from 40 weeks-old ApoE KO mice. Values shown are mean ± SD of all animals included in each group. *P < 0.05 *versus* TNFSF12^+/+^ApoE^−/−^; †*P* = 0.001 *versus* control IgG; white scale bar, 200 μm; black scale bar, 50 μm. (C) Plaque stability was assessed by forming α-SMA to Mac-3 ratio. Values shown are mean ± SD. *P < 0.05 *versus* TNFSF12^+/+^ApoE^−/−^; †*P* < 0.05 *versus* control IgG.

TNFSF12^−/−^ApoE^−/−^ and anti-TWEAK treated mice showed reduced lipid content compared with their respective control groups (Fig. 6A–C). Likewise, TNFSF12 deficiency and anti-TWEAK treatment also increased collagen content in brachiocephalic artery, although no statistically differences were observed (Fig. 6A–C). The collagen/lipid ratio of either TNFSF12^−/−^ApoE^−/−^ or anti-TWEAK treated mice was more than twice as high as in control groups (data not shown). No changes in VSMC content were observed between TNFSF12^−/−^ApoE^−/−^ or anti-TWEAK treated mice and control animals (Fig. 7A and B). Macrophage content was diminished in TNFSF12^−/−^ApoE^−/−^ or anti-TWEAK-treated mice compared with control animals (75% and 51%, respectively; *P* < 0.05 for both; Fig. 7A and B). As a consequence, VSMC/Macrophage ratio was increased in both TNFSF12^−/−^ApoE^−/−^ and anti-TWEAK treated mice (Fig. 7C), indicating an increment in the stability of these atherosclerotic lesions.

**Figure 8:**
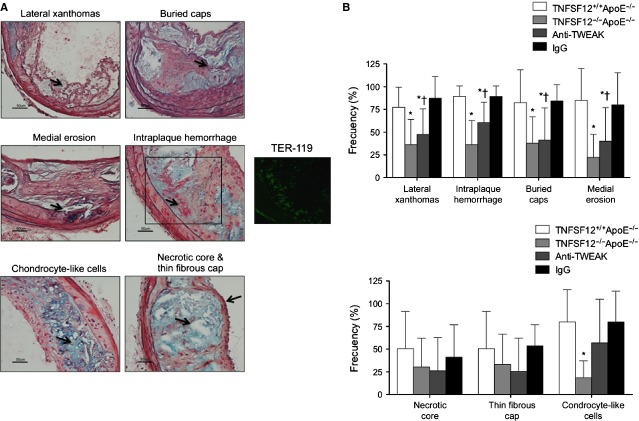
Tumour necrosis factor ligand superfamily member 12 (TNFSF12) deletion or anti-TNF-related weak inducer of apoptosis administration increased plaque stability in brachiocephalic artery of ApoE KO mice. (A) Representative photographs of Movat's pentachrome stained sections of the brachiocephalic artery of mice showing the presence of lateral xanthomas, buried fibrous caps, medial erosion, intraplaque haemorrhage (insert TER-119 staining), chondrocyte-like cells, necrotic core and thin fibrous cap; scale bar, 50 μm. (B) Frequency of morphologic markers of plaque instability in the brachiocephalic artery of ApoE KO mice. Values shown are the means ± SD of all animals included in each group. *P < 0.05 *versus* TNFSF12^+/+^ApoE^−/−^; †*P* < 0.05 *versus* control IgG.

Plaques were also analysed at the brachiocephalic artery for other features that both associate with and contribute to plaque instability such as the presence of lateral xanthomas, buried caps, necrotic core, thin fibrous cap, medial erosion, intraplaque haemorrhage and calcium content 13. Both TNFSF12^−/−^ApoE^−/−^ and anti-TWEAK-treated mice exhibited a reduced frequency of lateral xanthomas, medial erosion and presence of buried caps compared with TNFSF12^+/+^ApoE^−/−^ (Fig. 8A and B).

It has been suggested that intraplaque haemorrhage occur predominantly within the lateral xanthoma and is consistent with similar plaque rupture along the lateral margins in humans 13. The presence of intraplaque haemorrhage (defined as free red blood cells in the plaque matrix or filling the necrotic core) was diminished in both TNFSF12^−/−^ApoE^−/−^ and anti-TWEAK treated mice compared with control animals (Fig. 8A and B). The presence of intraplaque haemorrhage was confirmed by TER-119 immunostaining, a marker of red blood cells (Fig. 8A). The presence of red blood cells within atherosclerotic plaques is not related with neovessel formation since plaque vessels are not present in brachiocephalic arteries 13. Accordingly, we have not observed CD31^+^ cells within atherosclerotic plaques from brachiocephalic arteries of any group studied (data not shown).

Vascular smooth muscle cell can be converted to osteogenic (chondrocyte-like) cells *in vitro* suggesting that they play a role in vascular calcification *in vivo* 23, a marker of plaque vulnerability in humans 24. The presence of chondrocyte-like cells was diminished in TNFSF12^−/−^ApoE^−/−^ compared with TNFSF12^+/+^ApoE^−/−^ (Fig. 8). However, chondrocyte-like cells were non-significantly reduced in anti-TWEAK treated mice. However, both TNFSF12^−/−^ApoE^−/−^ and anti-TWEAK-treated mice exhibited a reduction in calcium content compared with controls (Fig. S9). Collectively, these results demonstrate that both TNFSF12 deficiency and anti-TWEAK treatment reduced several features of an unstable plaque phenotype.

### TNFSF12 deletion or anti-TWEAK treatment diminished gelatinolytic activity in advanced atherosclerotic lesions

**Figure 9:**
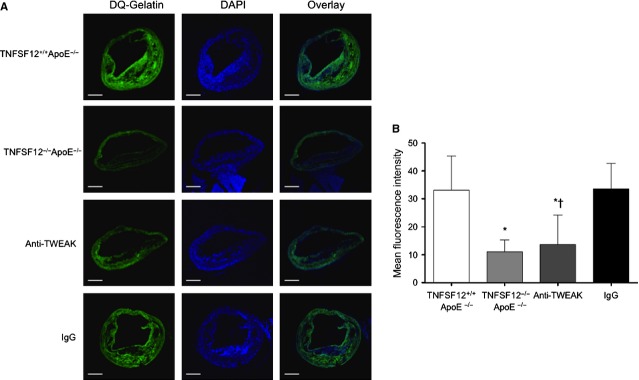
Tumour necrosis factor ligand superfamily member 12 (TNFSF12) deletion or anti-TNF-related weak inducer of apoptosis administration reduced gelatinase activity in brachiocephalic artery of ApoE KO mice. (A) Representative fluorescence photographs and (B) mean fluorescence intensity in the atherosclerotic lesions presents in the brachiocephalic artery assayed for *in situ* gelatinase activity. Values shown are mean ± SD of all animals included in each group. *P < 0.01 *versus* TNFSF12^+/+^ApoE^−/−^; †*P* < 0.05 *versus* control IgG; scale bar, 200 μm.

Besides plaque composition, MMP activity is thought to have a great impact on plaque stability 25. *In situ* zymography with quenched fluorescein-labelled gelatin in the atherosclerotic plaques present at the aortic root or brachiocephalic arteries showed reduced mean fluorescence intensity in TNFSF12^−/−^ApoE^−/−^ compared with TNFSF12^+/+^ApoE^−/−^ (Fig. S10 and Fig. 9, respectively). Anti-TWEAK treatment also reduced mean fluorescence intensity compared with their control mice. Pretreatment with two inhibitors of MMP activity, 0.5 mmol/l phenanthroline and 10 μmol/l ilomastat, or agarose gel lacking fluorescein-labelled gelatin substantially reduced fluorescence signal detected *via* 480-nm channel (Fig. S11). Taken together, these data provide evidence that TWEAK plays a role in vascular remodelling associated with atherosclerotic plaque instability.

## Discussion

Development, progression and ultimate rupture of an atherosclerotic plaque involve complex pathological mechanisms. Identification of underlying causes and successful medical treatment remains a major challenge in modern vascular medicine. Our results demonstrate that TWEAK participates in atherosclerotic plaque development, progression, and instability. TNFSF12 deletion or TWEAK blockade diminished atherosclerotic lesion size in both aortic root and brachiocephalic arteries of ApoE KO mice. Vascular smooth muscle cell were increased and macrophages and foam cells and gelatinolytic activity were reduced in atherosclerotic lesions of TNFSF12^−/−^ApoE^−/−^ or anti-TWEAK treated mice, effect associated with a decrease in the proinflammatory response. Interestingly, TNFSF12^+/−^ApoE^−/−^ mice also present a reduced atherosclerotic lesion size, macrophage content and proinflammatory cytokines expression indicating that partial inhibition of TWEAK expression limits atherosclerotic plaque progression. We provide evidence that TNFSF12 deletion or TWEAK blockade promotes multiple features of atherosclerotic plaque stability in brachiocephalic arteries, including a reduction in the presence of lateral xanthomas, buried caps, medial erosion, calcium content and intraplaque haemorrhage. Our results suggest that TWEAK blockade plays a dual atheroprotective role in advanced atherosclerotic plaques by promoting anti-inflammatory effects and enhancing features of plaque stability.

In agreement with previous studies in which TWEAK injection increased and treatment with Fn14-Fc fusion protein diminished atherosclerotic lesion size of ApoE KO mice 9,10, our results demonstrate that TNFSF12 deletion or blockade decreased atherosclerotic lesion size in mice. However, clinically, lesion composition rather than size or degree of stenosis of the lesion determine the likelihood of plaque rupture and subsequent thrombotic complications 2. Unstable plaques are rich in inflammatory cells and exhibit a substantial loss of VSMC and collagen content 26,27. TNFSF12 deletion or anti-TWEAK treatment induce a change in cellular composition in atherosclerotic plaques. The presence of VSMC was increased and macrophages infiltration was diminished in atherosclerotic lesions in the aortic root. In contrast, Schapira *et al*. observed a reduced number of VSMC and an increment in macrophages content after 12 weeks of treatment with Fn14-Fc in younger ApoE KO mice 10. These discrepancies could be due to the different strategies to block TWEAK/Fn14 interaction given that differential efficacy of other TNF inhibitors, the TNF-RII-Fc fusion protein and anti-TNF mAbs, has been observed 28. Another possible explanation is the existence of an alternative functional TWEAK receptor, suggesting that TWEAK may mediate some of its effects through receptors other than Fn14.

The underlying mechanism of the reduction in macrophage content by TNFSF12 deletion or anti-TWEAK treatment is most likely related to the reduction in chemokine expression. TWEAK increases the expression of several proinflammatory cytokines in cultured VSMC and macrophages, including MCP-1, IL-8, IL-6 and RANTES 9,29. These cytokines are controlled by the transcription factor NF-κB 20. We showed that prolonged inhibition of TWEAK signalling was able to maintain the reduction of chemokines expression and NF-κB activation, suggesting a chronic effect of TWEAK inhibition on inflammatory response observed in atherosclerotic lesions. We have also observed that circulating GM-CSF levels are diminished in double deficient or anti-TWEAK treated mice. GM-CSF is induced in endothelial cells by oxidized lipids and is present in atherosclerotic lesions 30, but its effects on plaque progression are controversial. Double deficient LDLR and GM-CSF mice exhibited 20–50% decrease in aortic lesion size 30. Similarly, ApoE KO mice treated with murine GM-CSF exacerbated atherosclerotic lesion area 31. In contrast, genetic deletion of GM-CSF in ApoE null mice increased lesion size 32. Furthermore, in agreement with our previous report 8, TF expression was diminished in either double deficient or anti-TWEAK treated mice, indicating that TWEAK is related with plaque thrombogenicity.

Lesional macrophages are a major source of MMP expression. We demonstrate herein a diminution in gelatinolytic activity in established atherosclerotic lesions from both TNFS12^−/−^ApoE^−/−^ or anti-TWEAK treated mice. Gelatinolytic activity is related to both MMP-2 and/or MMP-9 activation. Different authors have demonstrated that TWEAK is able to increase MMP-9 but not MMP-2 activity through NF-κB activation in several cell types including macrophages 29,33,34. In consequence, the mechanisms by which TNFSF12 deletion or anti-TWEAK treatment reduce gelatinolytic activity might be attributed to a diminution in macrophage content within atherosclerotic plaques but also to a reduction in NF-κB activity observed in our animal model. Degradation of collagen by MMPs has been demonstrated in human atherosclerotic lesions prone to rupture 35. Most likely as a direct consequence of the reduced infiltration or activation of MMP-secreting macrophages, the collagen content was ˜25% higher in advanced plaques of TNFSF12^−/−^ApoE^−/−^ than TNFSF12^+/+^ApoE^−/−^ mice. TNFSF12 deletion or anti-TWEAK treatment was capable of slowing down atheroprogression, strongly reducing ˜50% lipid deposits in advanced atherosclerotic plaques present in ApoE KO mice. This decrease is likely related to the reduction observed in both macrophages and foam cells, but could also be due to TWEAK's role in promoting lipid uptake by macrophages 10.

To further advance our understanding of the role of TWEAK in atherosclerotic plaque instability, we have analysed plaques present in brachiocephalic arteries of ApoE KO mice since phenotypic features of human plaque instability are more evident in unstable brachiocephalic atherosclerotic lesions than in aortic root 12. Vascular smooth muscle cell/macrophages and collagen/lipid ratios were increased and gelatinolityc activity was diminished in mice lacking TNFSF12 or treated with anti-TWEAK.

Moreover, TNFSF12 deletion or anti-TWEAK treatment reduces the presence of lateral xanthomas, aggregates of macrophage derived foam cells, which form fatty streaks along the margins of the established plaques 22, as well as buried caps, which may be the signature of silent plaque rupture. Formation of lateral fatty streaks will give rise to secondary lesions that layer on top of the established plaques, being one way by which the overall advanced plaque progresses 13. The disruption of these lateral xanthomas leads to intraplaque haemorrhage 13. In our study, 40 week-old ApoE KO mice present a high frequency of intraplaque haemorrhage (≈80%). This result is in agreement with data reported by Rosenfeld *et al*. who observed that up to 75% of ApoE KO mice aged 30–60 weeks exhibit intraplaque haemorrhage 13. TNFSF12 deletion or anti-TWEAK treatment reduced the presence of intraplaque haemorrhage in ApoE KO mice. Increased presence of red blood cells within plaques is associated with an unstable plaque phenotype in humans and may directly contribute to plaque instability owing to the high cholesterol content of red blood cells 36.

Another characteristic of unstable atherosclerotic plaques is the presence of large necrotic cores and thin fibrous caps that are predisposed to rupture 27. The frequency of necrotic core and thin fibrous cap in brachiocephalic arteries were diminished in TNFSF12^−/−^ApoE^−/−^ or anti-TWEAK treated mice compared with TNFSF12^+/+^ApoE^−/−^ or IgG treated mice although these differences did not achieve statistical significance. This could be due to the fact that in control mice the plaques with large necrotic cores progress, converting to highly fibrotic nodules. These nodules are highly cellular and most of these cells express markers of chondrocyte and osteoblast phenotype 37. This conversion from a plaque with a large necrotic zone to a fibrotic nodule is accompanied by conversion of resident VSMC to chondrocyte-like cells 23. Consistent with this notion, the chondrocyte-like cells were reduced in TNFSF12^−/−^ApoE^−/−^ and calcium content was diminished in both the TNFSF12^−/−^ApoE^−/−^ and anti-TWEAK treated mice. The formation of the fibrotic nodules is accompanied by the invasion of the medial layer by plaque leading to a thickening of the media (medial erosion) 22. We observed that the frequency of medial erosion was also reduced in both TNFSF12^−/−^ApoE^−/−^ or anti-TWEAK treated mice. Overall, these data indicate that TNFSF12 deletion or TWEAK blockade delay atheroprogression and increase plaque stability in ApoE KO mice.

In conclusion, our results not only support the notion that TWEAK plays a substantial role in atherosclerosis plaque formation and instability but also that blocking TWEAK-mediated pathway would be a new target for prevention and treatment of atherosclerosis. Blockade of TWEAK can recapitulate the genetic phenotype obtained in TNFSF12^−/−^ApoE^−/−^ preventing atherosclerotic complications by reduction of inflammatory response and associated macrophage recruitment and increasing plaque stability through an MMP-dependent mechanism.

## Supporting information

**Figure S1.** Schematic representation of the animal model. **Figure S2.** TNFSF12 deficiency or anti-TWEAK administration do not significantly alter metabolic parameters. **Figure S3.** TNFSF12 deletion decreased vascular damage in 24 weeks-old ApoE KO mice. **Figure S4.** TNFSF12 deletion diminished macrophage and foam cells and increased VSMC content in early atherosclerotic plaques from ApoE KO mice. **Figure S5.** TNFSF12 deletion diminished T cells content in advanced atherosclerotic plaques from ApoE KO mice. **Figure S6.** TNFSF12 deletion or anti-TWEAK administration did not modify proliferation or apoptosis in advanced atherosclerotic plaques of ApoE KO mice. **Figure S7.** TNFSF12 deletion diminished pro-inflammatory chemokine expression and NF-κB activation in early atherosclerotic plaques of ApoE KO mice. **Figure S8.** TNFSF12 deletion or anti-TWEAK administration diminished TF expression in advanced atherosclerotic plaques of ApoE KO mice. **Figure S9.** TNFSF12 deletion or anti-TWEAK administration diminished lesion calcification in brachiocephalic artery of ApoE KO mice. **Figure S10.** TNFSF12 deletion or anti-TWEAK administration reduced gelatinase activity in advanced atherosclerotic lesions of ApoE KO mice. **Figure S11.** MMP activity is inhibited by MMP inhibitors in the brachiocephalic artery of ApoE KO mice.
