# Supplementary material for: Genetic deletion or TWEAK blocking antibody administration reduce atherosclerosis and enhance plaque stability in mice
Source: J Cell Mol Med. 2014 Jan 30;18(4):721–34. doi: 10.1111/jcmm.12221 (PMC4000122; doi:10.1111/jcmm.12221)
Supplement: Supplementary file 1 — Figure S1. Schematic representation of the animal model. Figure S2. TNFSF12 deficiency or anti-TWEAK administration do not significantly alter metabolic parameters. Figure S3. TNFSF12 deletion decreased vascular damage in 24 weeks-old ApoE KO mice. Figure S4. TNFSF12 deletion diminished macrophage and foam cells and increased VSMC content in early atherosclerotic plaques from ApoE KO mice. Figure S5. TNFSF12 deletion diminished T cells content in advanced atherosclerotic plaques from ApoE KO mice. Figure S6. TNFSF12 deletion or anti-TWEAK administration did not modify proliferation or apoptosis in advanced atherosclerotic plaques of ApoE KO mice. Figure S7. TNFSF12 deletion diminished pro-inflammatory chemokine expression and NF-κB activation in early atherosclerotic plaques of ApoE KO mice. Figure S8. TNFSF12 deletion or anti-TWEAK administration diminished TF expression in advanced atherosclerotic plaques of ApoE KO mice. Figure S9. TNFSF12 deletion or anti-TWEAK administration diminished lesion calcification in brachiocephalic artery of ApoE KO mice. Figure S10. TNFSF12 deletion or anti-TWEAK administration reduced gelatinase activity in advanced atherosclerotic lesions of ApoE KO mice. Figure S11. MMP activity is inhibited by MMP inhibitors in the brachiocephalic artery of ApoE KO mice. [file jcmm0018-0721-sd1.pdf]

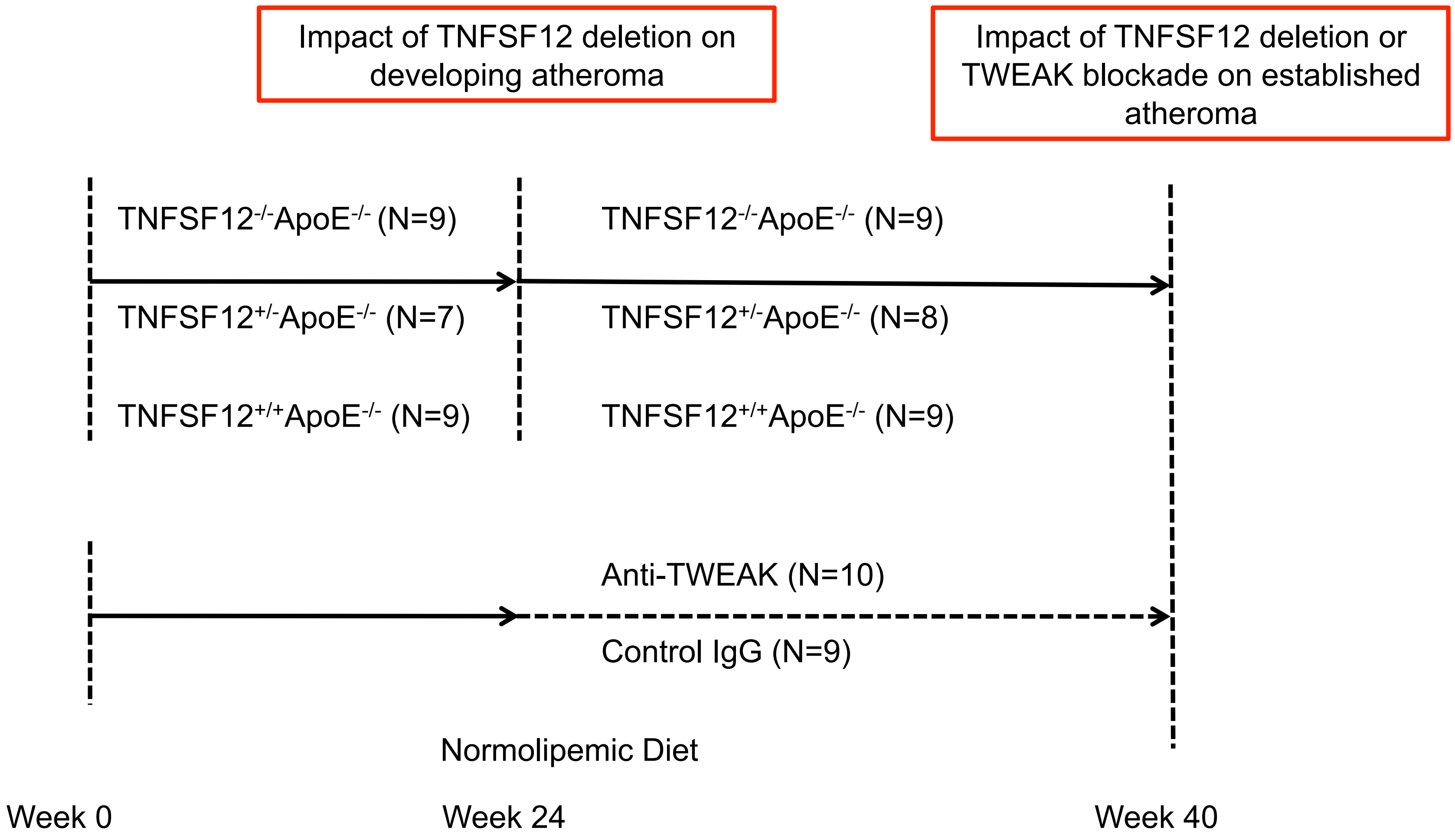
Impact of TNFSF12 deletion on developing atheroma
Impact of TNFSF12 deletion or TWEAK blockade on established atheroma
TNFSF12$^{-/-}$ApoE$^{-/-}$ (N=9)
TNFSF12$^{+/-}$ApoE$^{-/-}$ (N=7)
TNFSF12$^{+/+}$ApoE$^{-/-}$ (N=9)
TNFSF12$^{-/-}$ApoE$^{-/-}$ (N=9)
TNFSF12$^{+/-}$ApoE$^{-/-}$ (N=8)
TNFSF12$^{+/+}$ApoE$^{-/-}$ (N=9)
Anti-TWEAK (N=10)
Control IgG (N=9)
Normolipemic Diet
Week 0
Week 24
Week 40

Figure S1

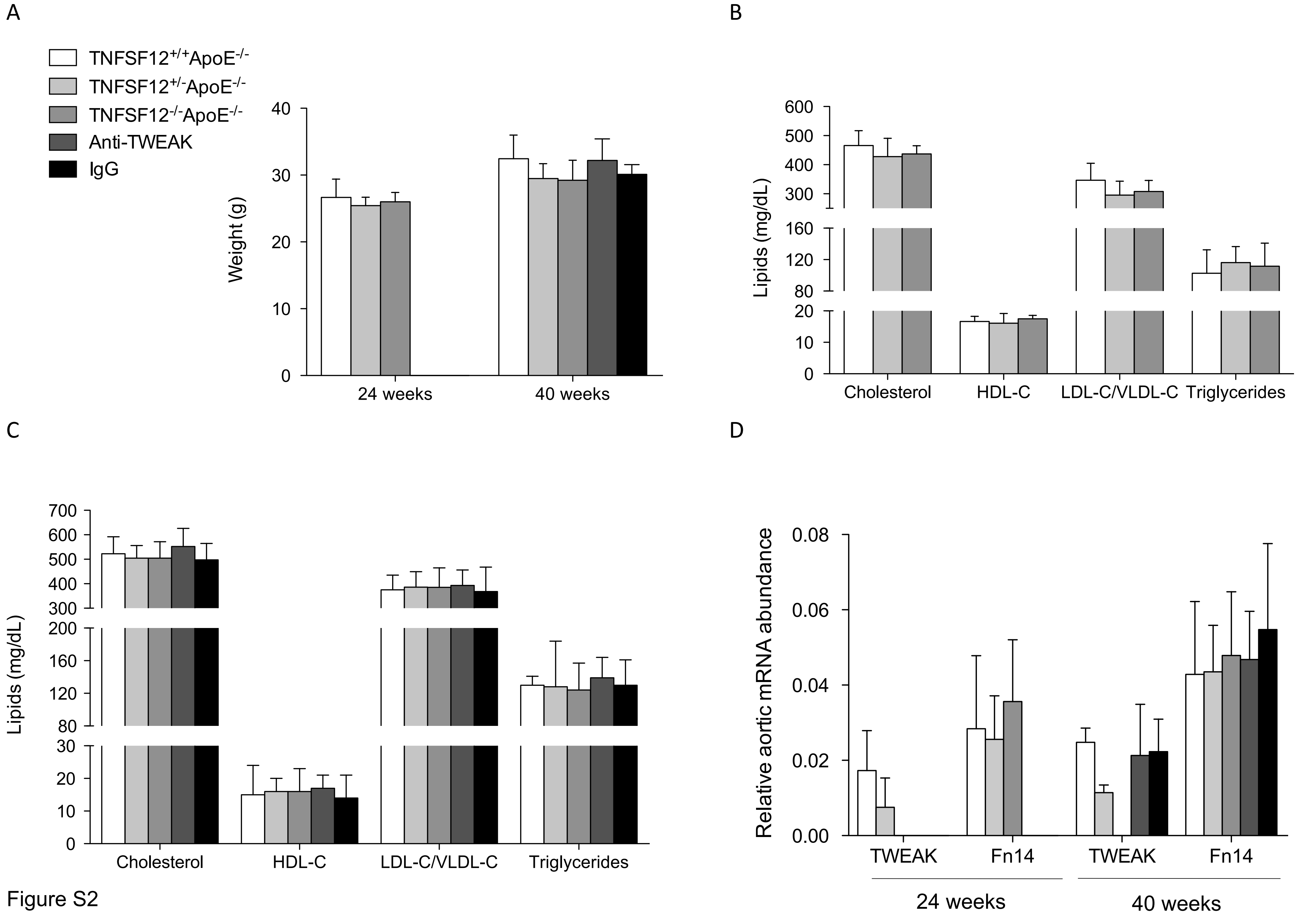
A
TNFSF12+/+ApoE-/-
TNFSF12+/-ApoE-/-
TNFSF12-/-ApoE-/-
Anti-TWEAK
IgG
Weight (g)
0
10
20
30
40
24 weeks
40 weeks
B
Lipids (mg/dL)
0
10
20
80
120
160
300
400
500
600
Cholesterol
HDL-C
LDL-C/VLDL-C
Triglycerides
C
Lipids (mg/dL)
0
10
20
30
80
120
160
200
300
400
500
600
700
Cholesterol
HDL-C
LDL-C/VLDL-C
Triglycerides
D
Relative aortic mRNA abundance
0.00
0.02
0.04
0.06
0.08
TWEAK
Fn14
TWEAK
Fn14
24 weeks
40 weeks

Figure S2

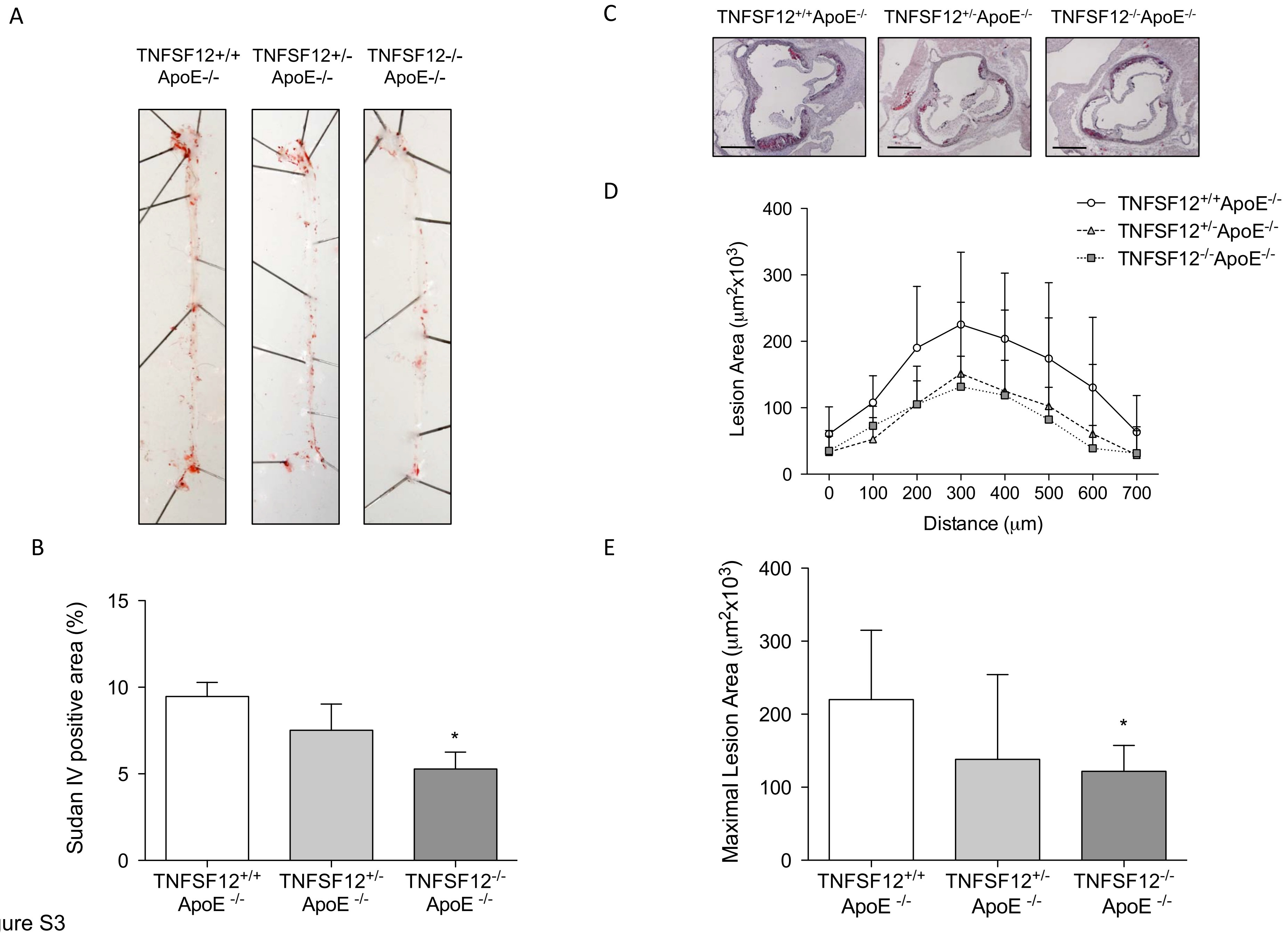
A
TNFSF12+/+ ApoE-/-
TNFSF12+/- ApoE-/-
TNFSF12-/- ApoE-/-
B
Sudan IV positive area (%)
15
10
5
0
*
TNFSF12+/+ ApoE -/-
TNFSF12+/- ApoE -/-
TNFSF12-/- ApoE -/-
C
TNFSF12+/+ApoE-/-
TNFSF12+/-ApoE-/-
TNFSF12-/-ApoE-/-
D
Lesion Area (μm2x103)
400
300
200
100
0
0
100
200
300
400
500
600
700
Distance (μm)
TNFSF12+/+ApoE-/-
TNFSF12+/-ApoE-/-
TNFSF12-/-ApoE-/-
E
Maximal Lesion Area (μm2x103)
400
300
200
100
0
*
TNFSF12+/+ ApoE -/-
TNFSF12+/- ApoE -/-
TNFSF12-/- ApoE -/-

Figure S3

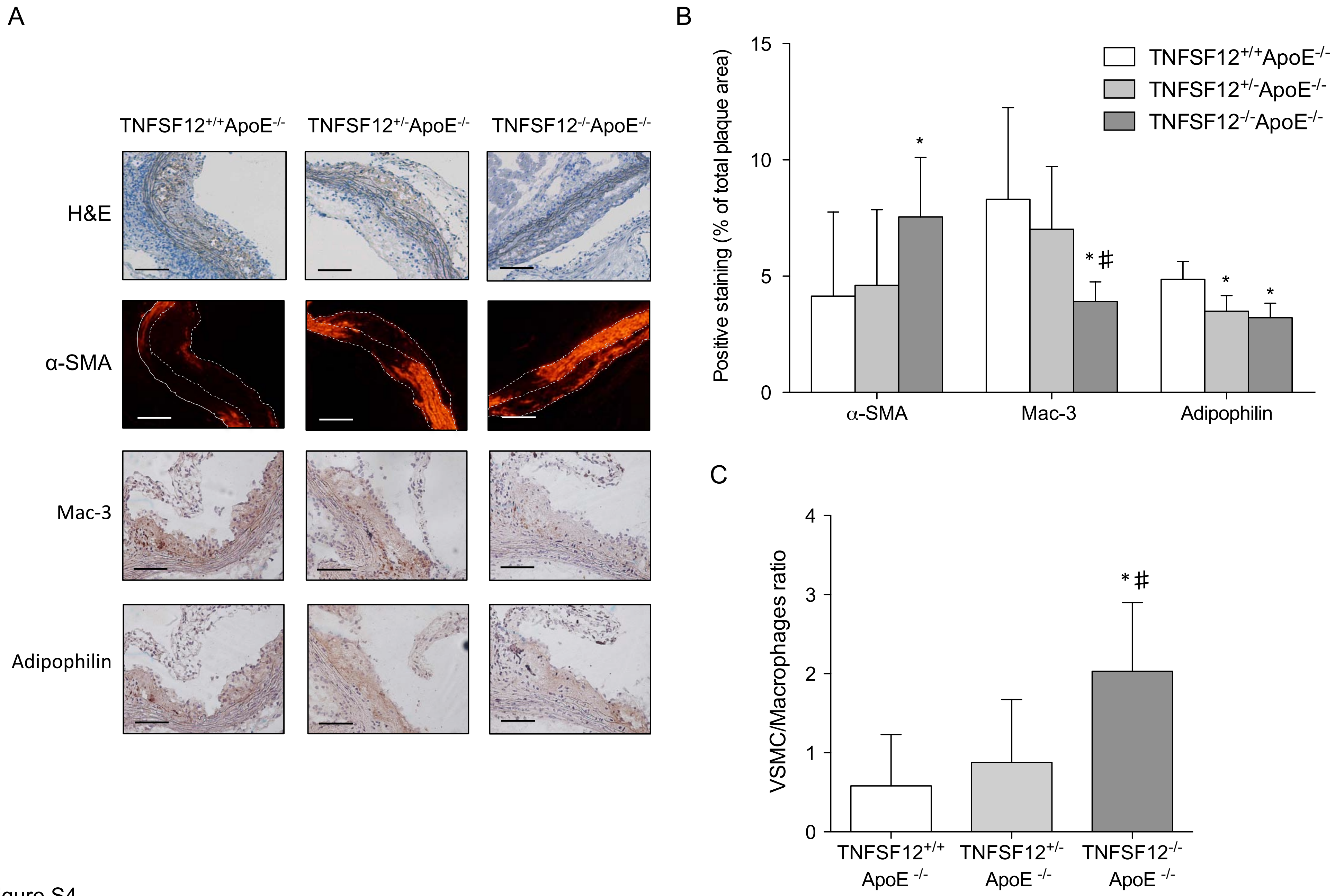
A
TNFSF12+/+ApoE-/-
TNFSF12+/-ApoE-/-
TNFSF12-/-ApoE-/-
H&E
α-SMA
Mac-3
Adipophilin
B
Positive staining (% of total plaque area)
15
10
5
0
TNFSF12+/+ApoE-/-
TNFSF12+/-ApoE-/-
TNFSF12-/-ApoE-/-
*
*#
*
*
α-SMA
Mac-3
Adipophilin
C
VSMC/Macrophages ratio
4
3
2
1
0
*#
TNFSF12+/+ ApoE -/-
TNFSF12+/- ApoE -/-
TNFSF12-/- ApoE -/-

Figure S4

A

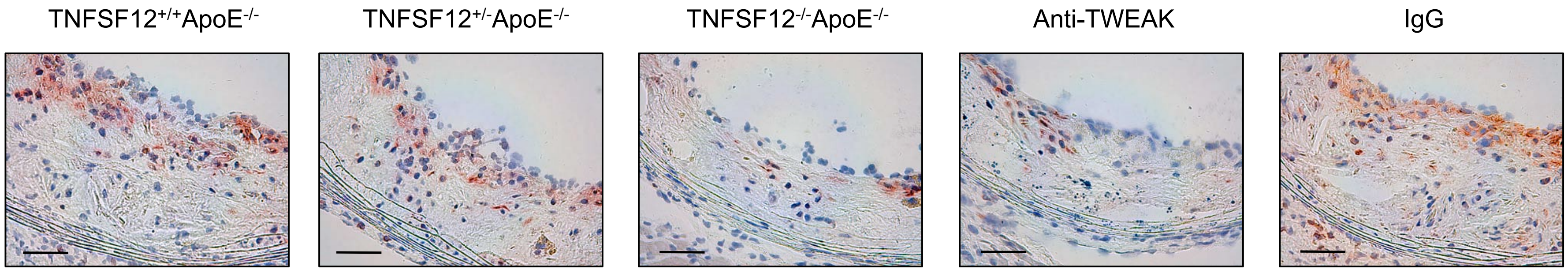

TNFSF12+/+ApoE-/-
TNFSF12+/-ApoE-/-
TNFSF12-/-ApoE-/-
Anti-TWEAK
IgG

B

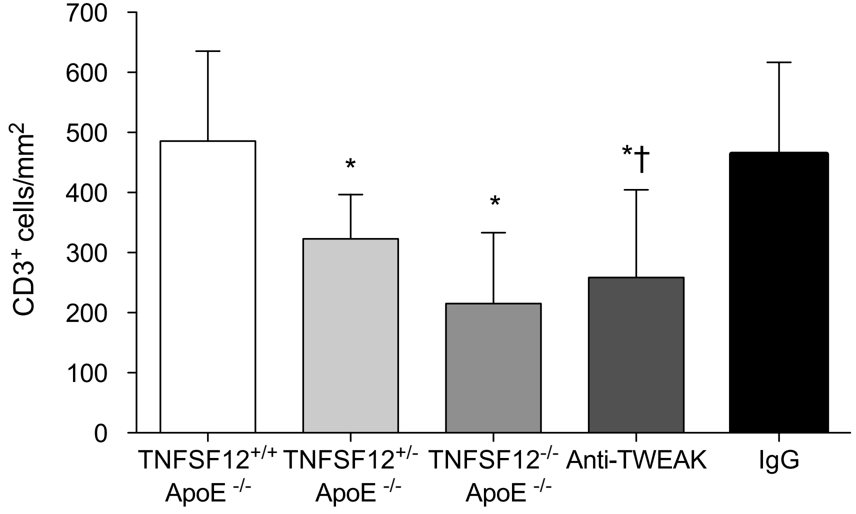

CD3+ cells/mm2
700
600
500
400
300
200
100
0
*
*
*†
TNFSF12+/+ ApoE -/-
TNFSF12+/- ApoE -/-
TNFSF12-/- ApoE -/-
Anti-TWEAK
IgG

Figure S5

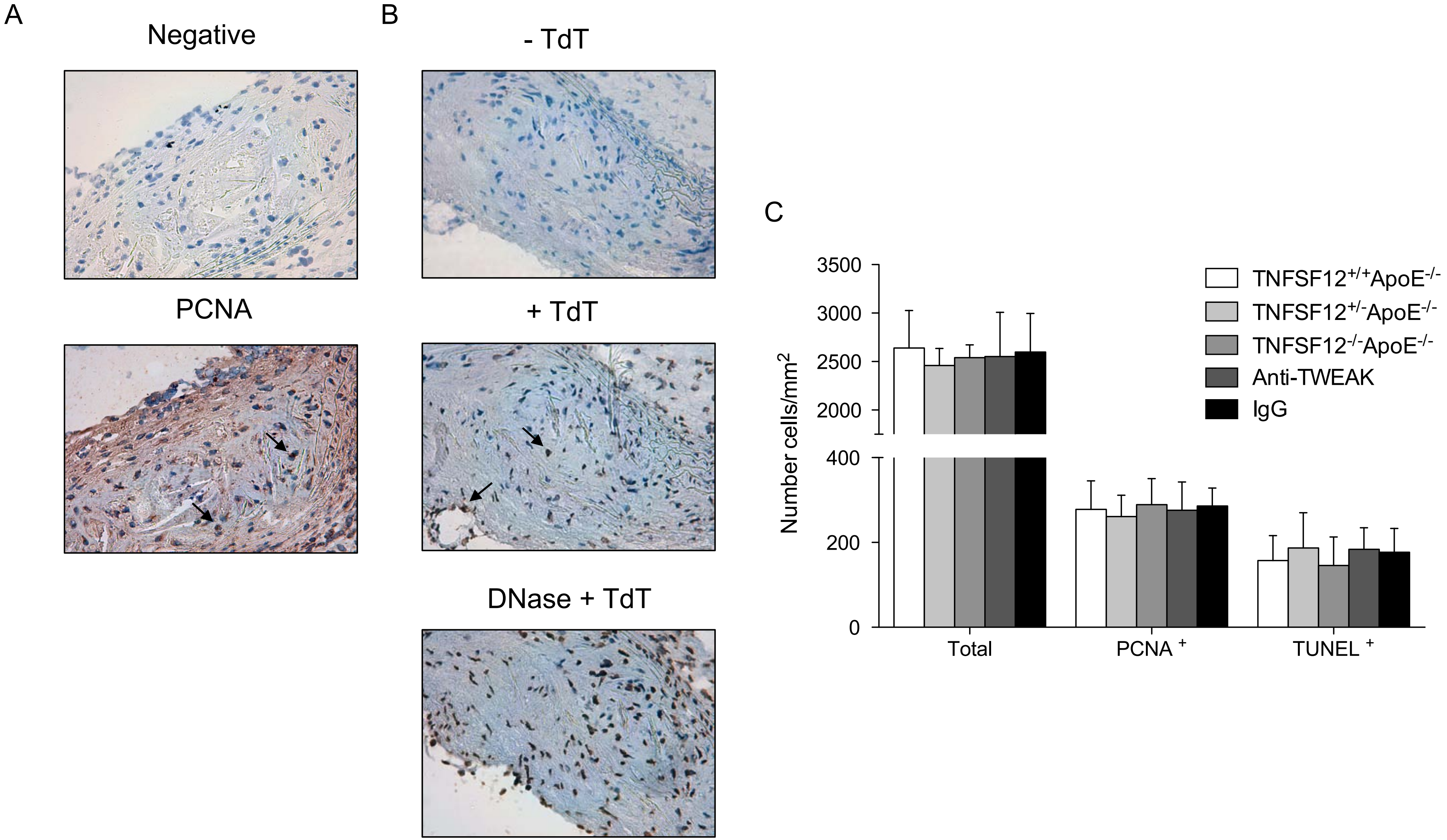
A
Negative
PCNA
B
- TdT
+ TdT
DNase + TdT
C
Number cells/mm2
3500
3000
2500
2000
400
200
0
Total
PCNA +
TUNEL +
TNFSF12+/+ApoE-/-
TNFSF12+/-ApoE-/-
TNFSF12-/-ApoE-/-
Anti-TWEAK
IgG

Figure S6

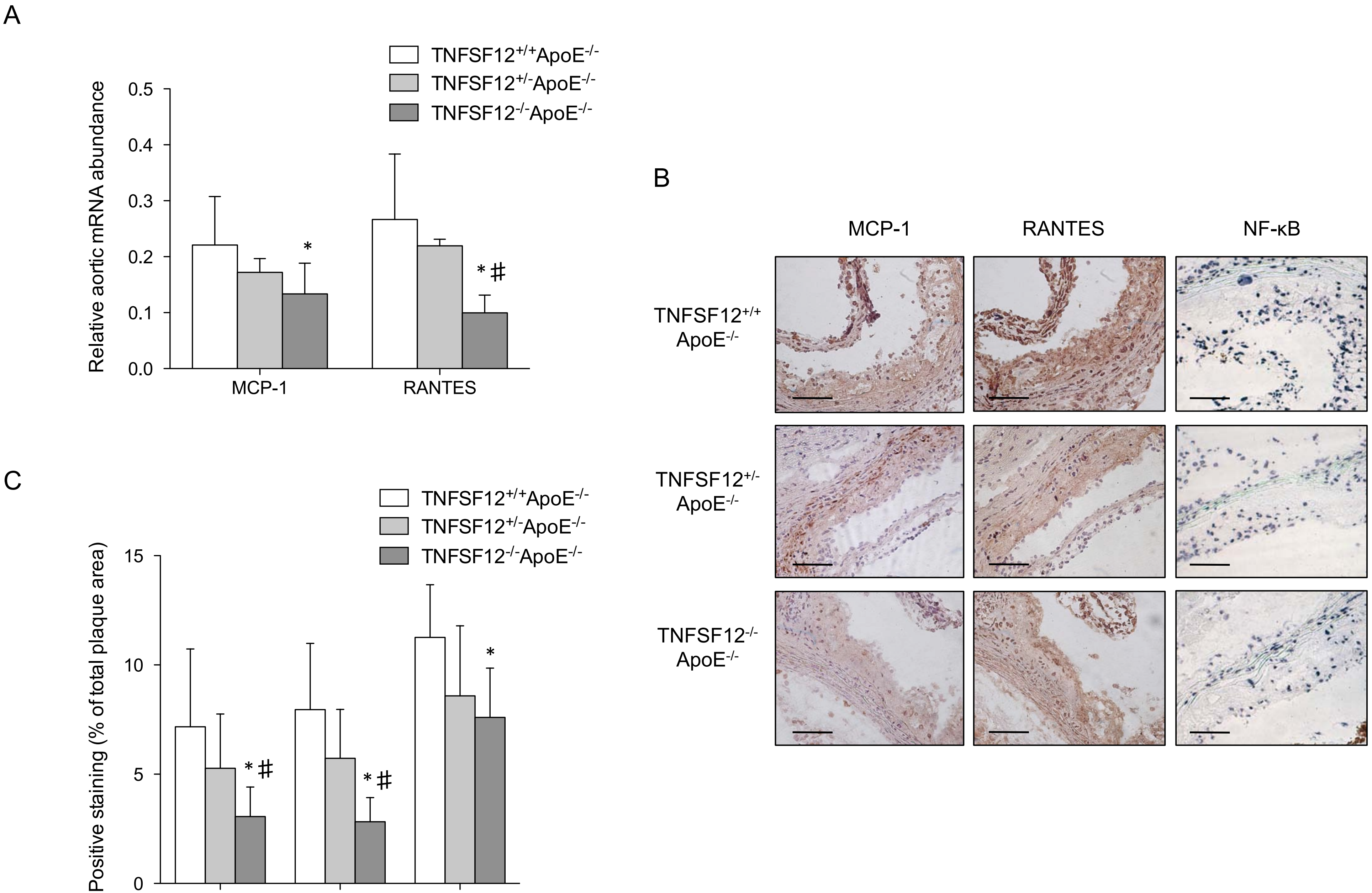
A
TNFSF12+/+ApoE-/-
TNFSF12+/-ApoE-/-
TNFSF12-/-ApoE-/-
Relative aortic mRNA abundance
0.5
0.4
0.3
0.2
0.1
0.0
MCP-1
RANTES
B
MCP-1
RANTES
NF-κB
TNFSF12+/+ ApoE-/-
TNFSF12+/- ApoE-/-
TNFSF12-/- ApoE-/-
C
TNFSF12+/+ApoE-/-
TNFSF12+/-ApoE-/-
TNFSF12-/-ApoE-/-
Positive staining (% of total plaque area)
15
10
5
0
MCP-1
RANTES
NF-κB

Figure S7

A

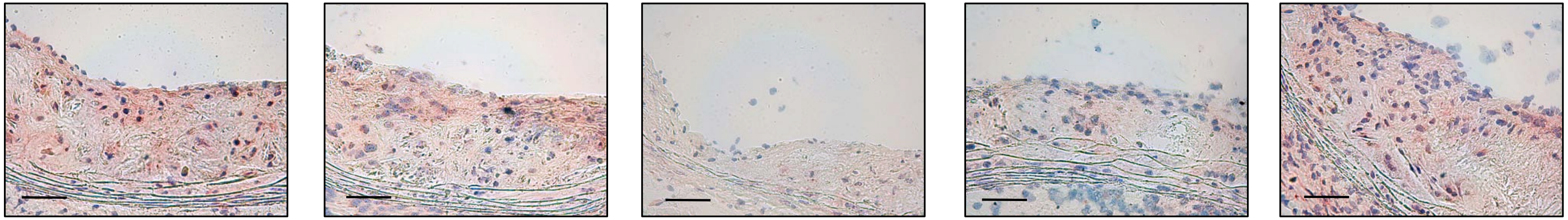

TNFSF12+/+ApoE-/-
TNFSF12+/-ApoE-/-
TNFSF12-/-ApoE-/-
Anti-TWEAK
IgG

B

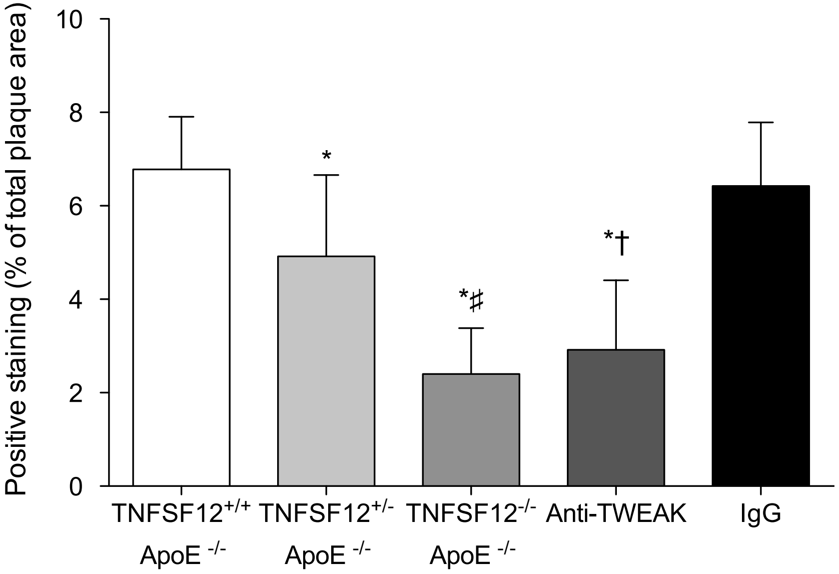

Positive staining (% of total plaque area)
10
8
6
4
2
0
*
*♯
*†
TNFSF12+/+ ApoE -/-
TNFSF12+/- ApoE -/-
TNFSF12-/- ApoE -/-
Anti-TWEAK
IgG

Figure S8

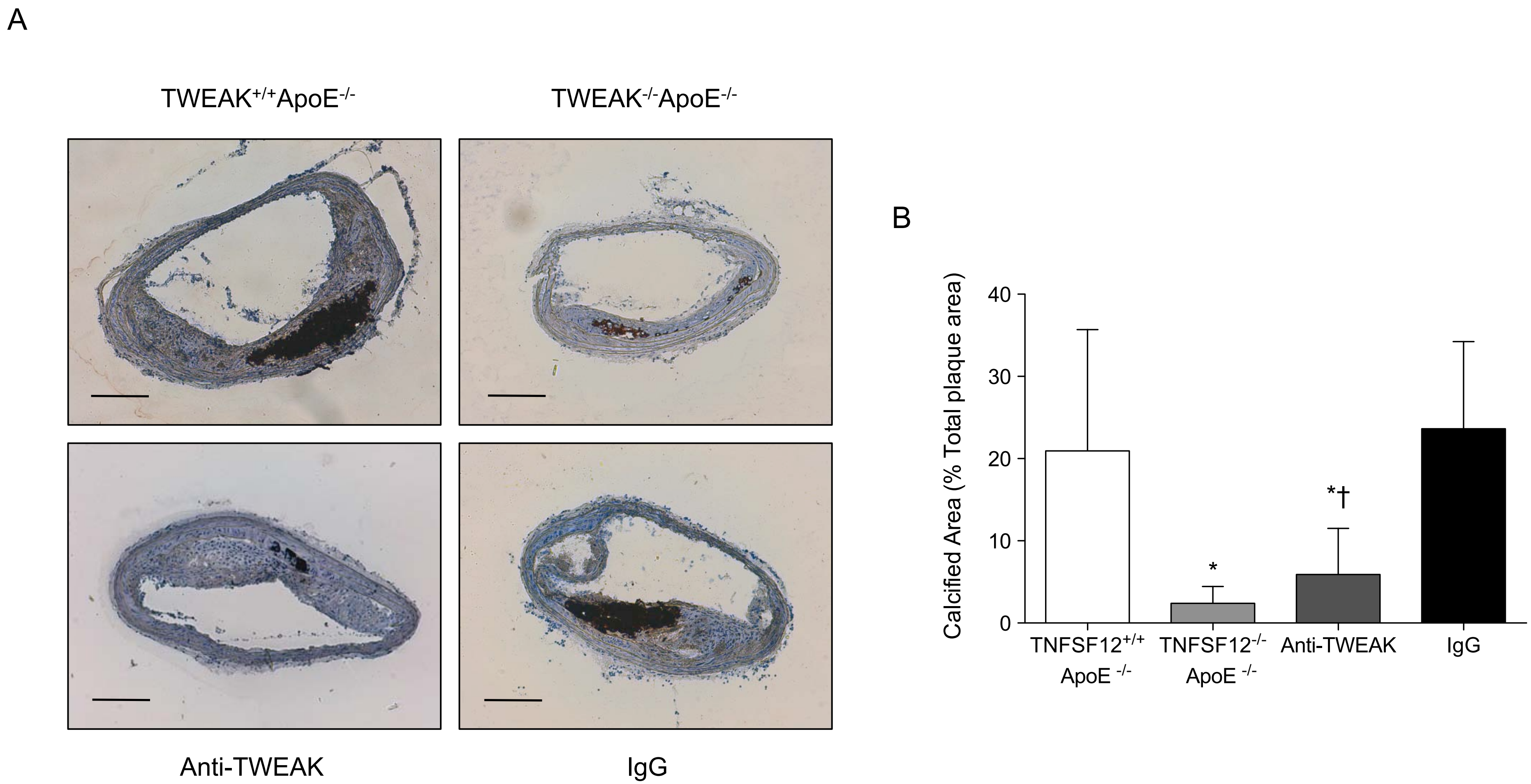
A
TWEAK+/+ApoE-/-
TWEAK-/-ApoE-/-
Anti-TWEAK
IgG
B
Calcified Area (% Total plaque area)
40
30
20
10
0
*
*†
TNFSF12+/+ ApoE -/-
TNFSF12-/- ApoE -/-
Anti-TWEAK
IgG

Figure S9

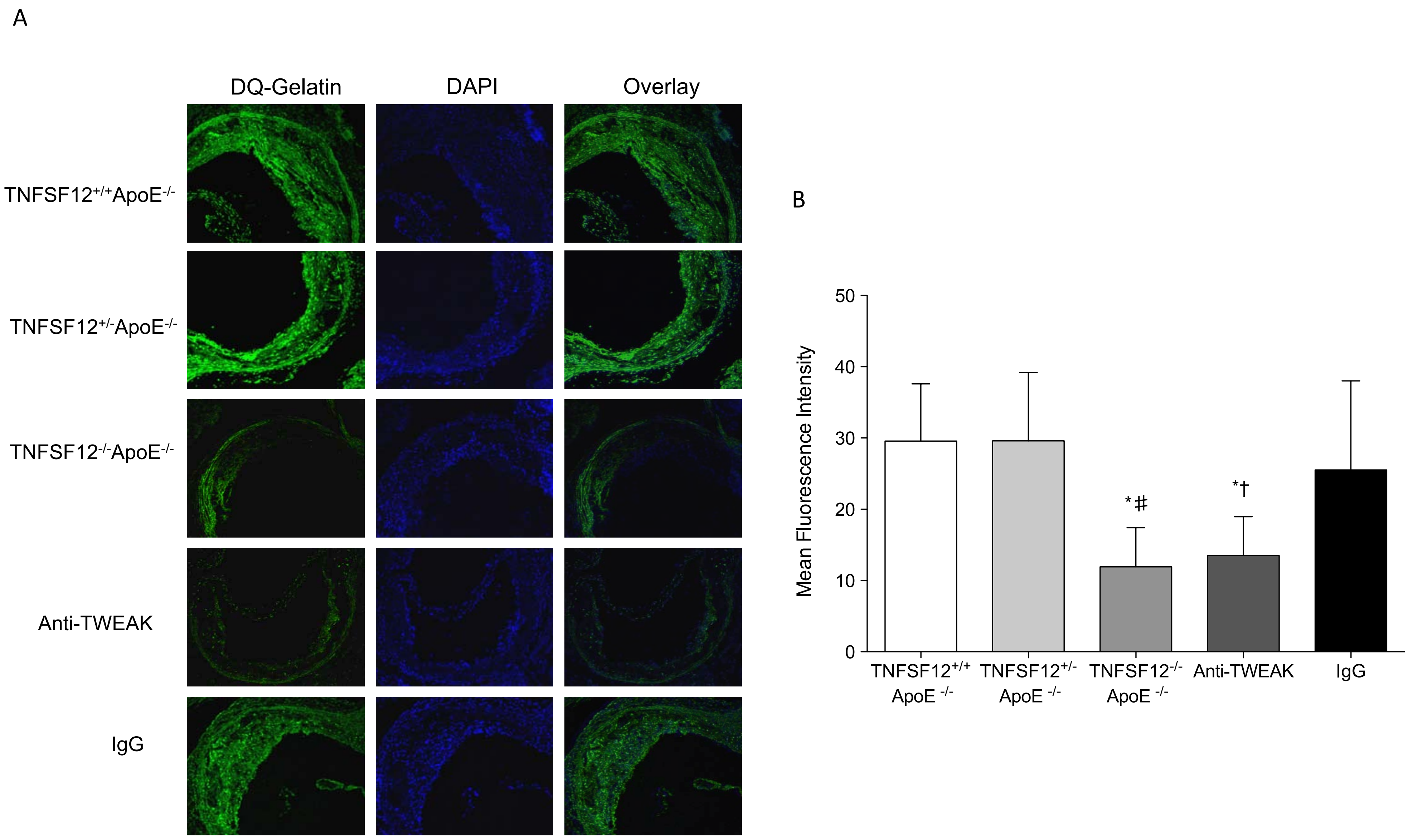
A
DQ-Gelatin
DAPI
Overlay
TNFSF12+/+ApoE-/-
TNFSF12+/-ApoE-/-
TNFSF12-/-ApoE-/-
Anti-TWEAK
IgG
B
Mean Fluorescence Intensity
0
10
20
30
40
50
*#
*†
TNFSF12+/+ ApoE -/-
TNFSF12+/- ApoE -/-
TNFSF12-/- ApoE -/-
Anti-TWEAK
IgG

Figure S10

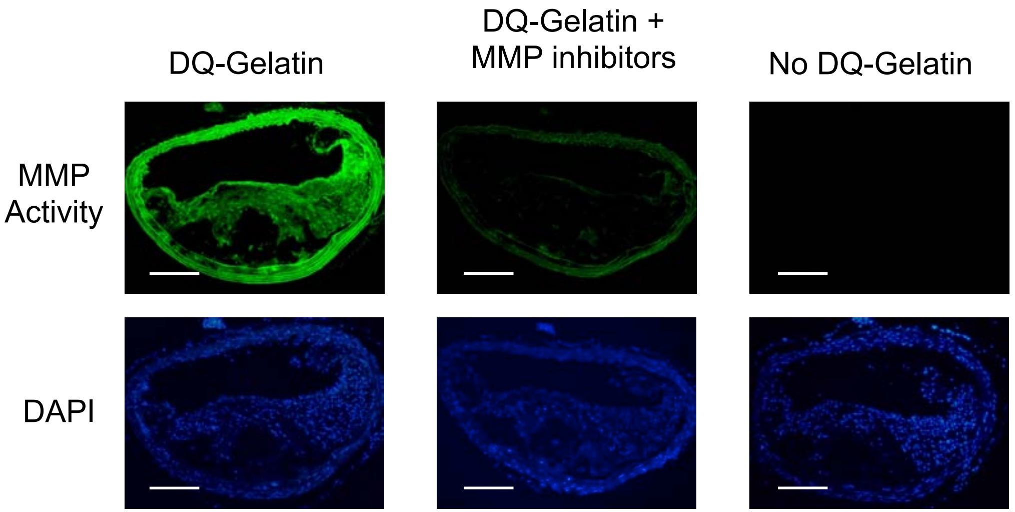
DQ-Gelatin
DQ-Gelatin +
MMP inhibitors
No DQ-Gelatin
MMP
Activity
DAPI

Figure S11
